# CDKN2A/B deletions are strongly associated with meningioma progression: a meta-analysis of individual patient data

**DOI:** 10.1186/s40478-023-01690-y

**Published:** 2023-11-28

**Authors:** Johannes Wach, Alim Emre Basaran, Felix Arlt, Martin Vychopen, Clemens Seidel, Alonso Barrantes-Freer, Wolf Müller, Frank Gaunitz, Erdem Güresir

**Affiliations:** 1 Department of Neurosurgery, University Hospital Leipzig, 04103 Leipzig, Germany; 2 Department of Radiation Oncology, University Hospital Leipzig, 04103 Leipzig, Germany; 3 Department of Neuropathology, University Hospital Leipzig, 04103 Leipzig, Germany

**Keywords:** CDKN2A/B, Individual patient data, Meningioma, Meta-analysis, Progression-free survival

## Abstract

Homozygous CDKN2A/B deletion has been associated with an increased risk of recurrence in meningiomas. However, the evidence is confined to a limited number of studies, and the importance of heterozygous CDKN2A/B deletions remains insufficiently investigated. Hence, the present meta-analysis reconstructs individual patient data (IPD) and reconstructs the probabilities of progression-free survival (PFS) stratified by CDKN2A/B status. IPD of PFS rates were extracted from published Kaplan–Meier plots using the R package *IPDfromKM* in R studio (RStudio, Boston, MA, USA). Reconstructed Kaplan–Meier Plots of the pooled IPD data were created. One-stage and two-stage meta-analyses were performed. Hazard ratios (HR) were used as effective measures. Of 181 records screened, four articles with 2521 participants were included. The prevalence of homozygous CDKN2A/B deletions in the included studies was 0.049 (95% CI 0.040–0.057), with higher tumor grades associated with a significantly greater proportion of CDKN2A/B deletions. The reconstructed PFS curves for the pooled cohort showed that the median PFS time of patients with a CDKN2A/B wild-type status, heterozygous or homozygous CDKN2A/B deletion was 180.0 (95% CI 145.7–214.3), 26.1 (95% CI 23.3–29.0), and 11.00 (95% CI 8.6–13.3) months, respectively (*p* < 0.0001). Both hetero- or homozygous CDKN2A/B deletions were significantly associated with shortened time to meningioma progression. One-stage meta-analysis showed that hetero- (HR: 5.5, 95% CI 4.0–7.6, *p* < 0.00001) and homozygous CDKN2A/B deletions (HR: 8.4, 95% CI 6.4–11.0, *p* < 0.00001) are significantly associated with shortened time to meningioma progression. Multivariable Cox regression analysis of progression in a subgroup with available covariates (age, sex, WHO grade, and TERT status) and also two-stage meta-analysis confirmed and validated the results of the one-stage analysis that both heterozygous and homozygous CDKN2A/B deletions are of prognostic importance. Further large-scale studies of WHO grade 2 and 3 meningiomas are needed to validate the importance of heterozygous CDKN2A/B deletions with consideration of established factors.

**Supplementary Information:**

The online version contains supplementary material available at 10.1186/s40478-023-01690-y.

## Introduction

Meningiomas represent the most prevalent type of intracranial tumor among adults [27]. Although the majority of meningiomas are benign World Health Organization (WHO) grade 1 tumors, approximately 20–30% are WHO grade 2 tumors with aggressive behavior and exhibit tumor progression despite undergoing cytoreductive surgery and radiation treatment [39, 43].

At present, these clinical factors are incorporated into the WHO grading of meningiomas, a process predominantly dependent on histopathological evaluation [20]. When comparing aggressive meningiomas with their counterparts, the former exhibit a higher incidence of copy number variations, including specific deletions [26]. Notably, homozygous cyclin-dependent kinase inhibitor 2A/B (CDKN2A/B) gene deletions of the chromosome 9p21 have been linked to a significantly shortened time to meningioma progression and has been recommended as diagnostic characteristics by the WHO grading since the last revision of the classification system [20]. Nevertheless, the presence of CDKN2A/B deletions is assumed to be rare, with rates being reported between 1.7 and 6.7% [9]. Despite there are very strong data supporting the prognostic importance of homozygous CDKN2A/B deletions in terms of meningioma progression, the impact of heterozygous CDKN2A/B deletions on progression-free survival (PFS) is not entirely clear so far.

Against this backdrop, there is the need to evaluate the pooled prevalence and clinical effect of this gene deletion in meningiomas. The present meta-analysis investigates the clinical prevalence of CDKN2A/B deletions and the impact of heterozygous or homozygous CDKN2A/B deletions on PFS.

## Methods and materials

This meta-analysis was conducted according to the Preferred Reporting Items for Systematic Reviews and Meta-analyses (PRISMA) reporting guideline for individual patient data (IPD) development cohorts [38], and the study protocol was prospectively submitted in the “International Prospective Register of Systematic Reviews” (ID: 455131). Since the present meta-analysis relied on previously published studies, there was no need for ethical approval from the institutional ethics committee or informed consent from patients.

### Search strategy and study inclusion

We searched three databases, PubMed, Web of Science, and Cochrane library, for all investigations regarding CDKN2A/B deletion in meningiomas up to June 30, 2023. Studies published in English were retrieved. The search strategy was performed based on the PICOS criteria [34]. The following mesh terms were used to search for eligible studies: (1) “meningioma” AND “CDKN2A”; (2) “meningioma” AND “CDKN2B”; (3) “meningioma” AND “CDKN2A/B”. The search strategy adhered to the Preferred Reporting Items for Systematic Reviews and Meta-Analyses (PRISMA) guidelines [28]. Inclusion criteria required CDKN2A/B status in meningioma and follow-up data regarding PFS. Identified studies were excluded if either CDKN2A/B status or PFS data was not available. Two reviewers (JW, AB) independently screened abstracts, and full-text articles for two rounds, with any residual conflicts resolved by a third reviewer (EG).

### Quality assessment

The National Institutes of Health Quality Assessment Tool for observational cohort and cross-sectional studies (NIH-QAT) was used for the assessment of quality and risk of bias of included studies [23].

### Data extraction

Two authors (JW, AB) independently extracted the following data from the publications: clinical and neuropathological characteristics of meningioma cases, prevalence of homozygous or heterozygous CDKN2A/B deletion, and PFS data in cases with or without homozygous/heterozygous CDKN2A/B deletion. The individual patient data information of PFS was extracted from the published Kaplan–Meier survival curves and number at risk tables using DigitizeIt (Version 2.5.10 for macOS, Braunschweig, Germany) [32]. The complete IPD of the study by Sievers et al. [37], and the IPD of the patients with CDKN2A/B deletions of the study by Khan et al. [16] were extracted from the supplementary materials of these manuscripts. Additionally, the IPD of Khan et al. [16] and Sievers et al. [37] enabled a further analysis of PFS data stratified by both WHO grade and CDKN2A/B status. This procedure was performed for meningioma patients with Wild-type as well as heterozygous or homozygous CDKN2A/B deletion. The extracted progression-free survival data and the published number at risk tables were used to reconstruct the Kaplan–Meier curves for each included study using the method of Liu et al. [19] using the R package *IPDfromKM* in R studio (RStudio, Boston, MA, USA). The risk tables were also generated. We compared the reconstructed curves, risk tables, estimated HRs, and estimated 95% confidence intervals (CI) with those in the original publications. The extraction of information was repeated if there were apparent discrepancies.

### Statistical analysis, one- and two-stage meta-analysis with individual patient data

Patient demographics and disease-specific characteristics of the included studies were recorded and compared using the two-sided Pearson’s chi-squared test. The prevalence of meningioma patients with CDKN2A/B status among those with available PFS data as well as among the entire cohorts stratified by WHO grades were determined for each study and were analyzed using the R based software OpenMeta-Analyst, using the *Metafor* R package [41, 45]. Data was pooled and effect size calculated using a random-effects model considering the weight of each individual investigation. Heterogeneity in the estimated prevalence of CDKN2A/B deletion across the included studies was evaluated using I^2^ statistics, with a threshold of > 50% indicating substantial heterogeneity [11].

The IPD information of all time-to-meningioma progression data from all the included studies was combined, and Kaplan–Meier plots of PFS were constructed using the R package *Survminer* and *Survival* in R software version 4.3.1 (R Foundation for Statistical Computing, Vienna, Austria). The 1-, 2-, and 5-years progression-free survival rates were constructed. The hazard ratios (HR) of each included study as well as the overall HR and 95% CI between meningioma patients with CDKN2A/B deletion and wild-type CDKN2A/B were estimated. Subgroup analysis of 1185 patients with PFS data with stratification of CDKN2A/B status by WHO grade was performed for the cohorts by Khan et al. [16] and Sievers et al. [37]. Two-hundred-eighty-seven patients of this subgroup also shared the common available covariates age, sex, WHO grade, TERT promoter status, and CDKN2A/B status. Multivariable cox regression analysis of factors predicting meningioma progression was performed among this subgroup. Visualization of the results from the multivariable Cox regression analysis was supported by Prism 8 for macOS (Version 8.4.3, GraphPad Software, San Diego, CA, USA). In the two-stage meta-analysis the estimated HRs and the corresponding 95% CIs of the individual studies were pooled using a random-effects model with the generic inverse variance method. The estimated hazard ratios were converted to the natural logarithm (LN). The standard errors (SE) of each study was calculated form the 95% CI using the following formula: SE = (LN (upper CI limit) − LN (lower CI limit))/3.92 (in line with the Cochrane Handbook for Systematic Reviews of Interventions, Version 6.4) [12]. Weight of the relative contribution of each study, based on the sample size, was considered regarding the estimation of the treatment effects. The pooled estimates were displayed in forest plots using the Review Manager Web (RevMan Web Version 5.4.1 from The Cochrane Collaboration). *p* < 0.05 was considered as statistically significant. Publication bias was visually assessed using funnel plots with the R package *Meta*. Furthermore, the likelihood of publication bias was statistically investigated. Egger’s regression test was applied to investigate the publication bias and a 5% significance threshold was set [7]. The publication bias was assessed using the R package *metafor*.

## Results

### Study selection and study characteristics

**Fig. 1:**
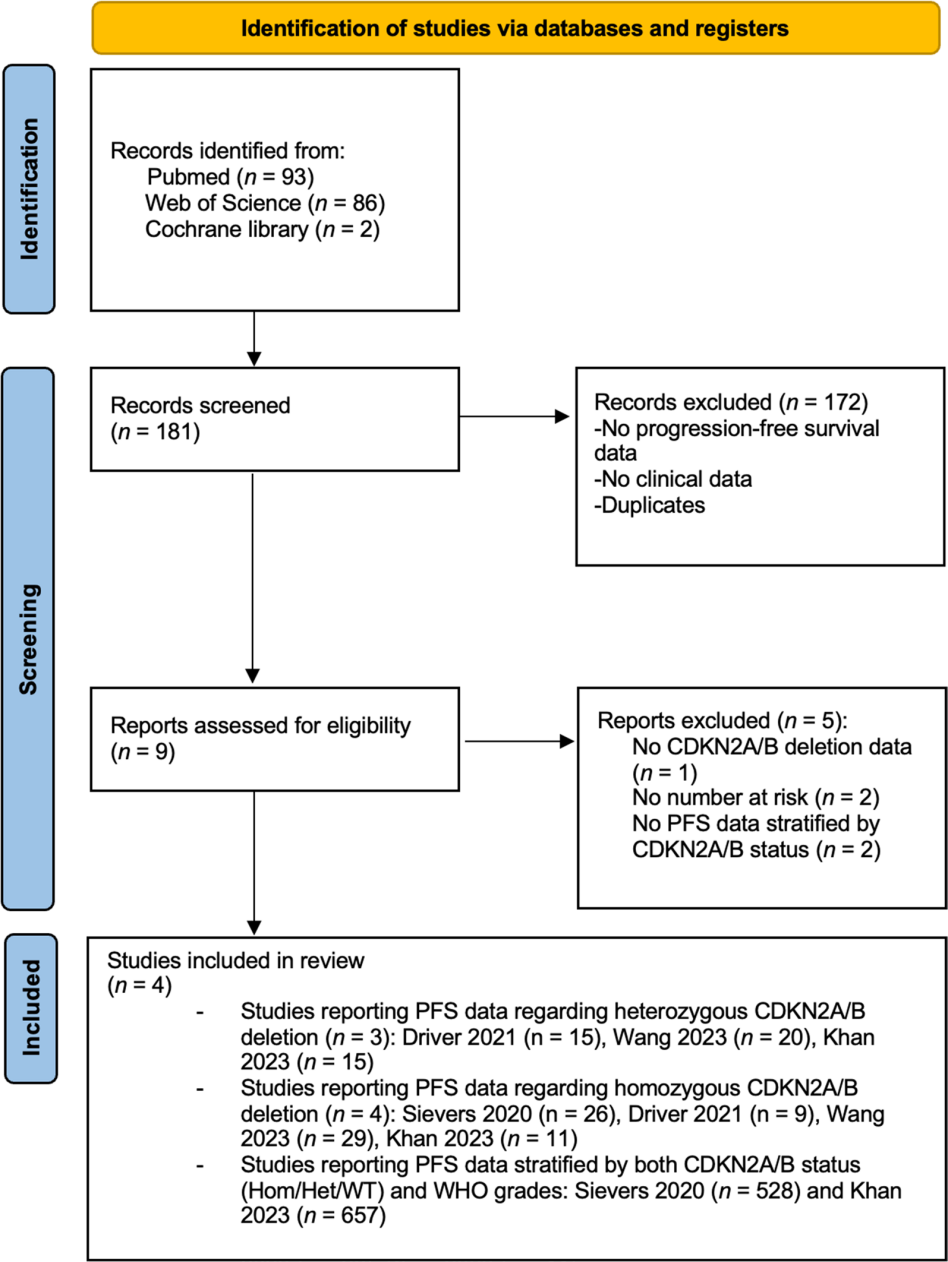
PRISMA flowchart for study selection

We identified 181 records with four investigations meeting inclusion criteria (Fig. 1) [6, 16, 37, 46].

A total of 2521 patients with both CDKN2A/B data and PFS data were identified. The studies included in this analysis are authored by Sievers et al. [37], Khan et al. [16]. Wang et al. [46], and Driver et al. [6]. The DKFZ cohort of the study by Wang et al. [46] was not included in the PFS analysis because of potential overlap with IPD from Sievers et al. [37]. The clinical and pathological characteristics were described and compared across the studies. All studies showed a female predominance and there was no heterogeneous distribution regarding sex. The median age (58.5), along with the interquartile range (IQR) (48.0–67.0), is reported for the study by Sievers et al. [37]. The mean age (59) and range (9–90) are given for the study by Driver et al. [6]. No information regarding age is available for the study by Wang et al. [46]. The extent of resection is categorized into gross total resection (GTR) and subtotal resection (STR) in the studies by Driver et al. [6] and Wang et al. [46]. A statistically significant difference (*p* = 0.013) is noted regarding extent of resection between the studies by Driver et al. [6] and Wang et al. [46]. The anatomical location of meningiomas is only reported in the study by Sievers et al. [37]. The most common location was at the convexity (58.3%). The presence of TERT promoter mutation is reported in the studies by Sievers et al. [37] and Driver et al. [6]. Pearson’s chi-squared test showed no significant difference regarding the proportions of TERT promoter mutations among both studies. Further details are summarized in Table 1.

**Table 1:** Distribution of clinical and pathological patient characteristics among the included studies

Parameters	Sievers et al. [37]	Wang et al. [46]	Driver et al. [6]	Khan et al. [16]	*p*
Sex					0.45
Female	350/528	698/1059 (65.9%)	353/527 (67.0%)	NA (only available among those with CDKN2A/B deletions)	
Male	(66.28%)	391/1059 (36.9%)	174/527 (33.0%)		
	178/528 (33.71%)				
Age (in years)	58.5 (median) (IQR: 48.0–67.0)	NA	57 (mean) (range: 9–90)	NA (only available among those with CDKN2A/B deletions)	
Extent of resection				NA (only available among those with CDKN2A/B deletions)	0.013
GTR	NA	418/588 (71.1%)	338/527 (64.1%)		
STR	NA	170/588 (28.9%)	189/527 (35.9%)		
Location					
Skull base	95/528 (18.0%)	NA	NA	NA	
Convexity	308/528 (58.3%)				
Posterior fossa	38/528 (7.2%)				
Spinal					
Supratentorial	27/528 (5.1%)				
NA	34/528 (6.4%)				
	24/528 (4.6%)				
TERT promoter mutation	6/293 (2.05%)	NA	6/244 (2.46%)	NA (only available among those with CDKN2A/B deletions)	0.75

GTR, gross total resection; IQR, interquartile range; NA, not available; STR, subtotal resection; TERT, telomerase reverse transcriptase

### Prevalence of CDKN2A/B deletion

**Fig. 2:**
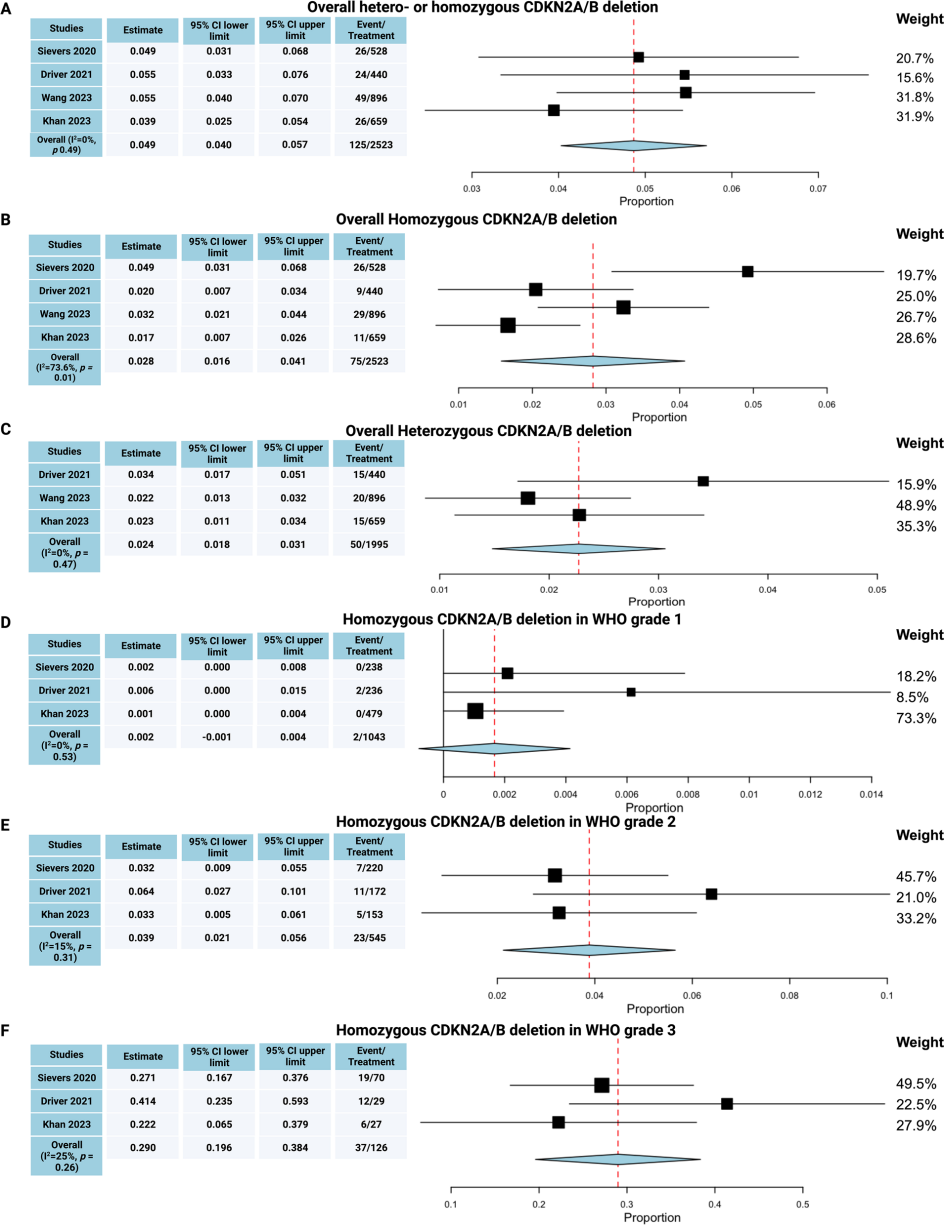
**a** Overall prevalence of homozygous and heterozygous CDKN2A/B deletions in meningioma. **b** Prevalence of homozygous CDKN2A/B deletions in meningioma. **c** Prevalence of heterozygous CDKN2A/B deletions in meningioma. Prevalence of homozygous CDKN2A/B deletions in WHO grade 1 (**d**), 2 (**e**), and 3 (**f**) meningiomas

Across the four included investigations regarding meningioma patients with CDKN2A/B status and available PFS data, the pooled prevalence of CDKN2A/B deletion (hetero- or homozygous) was 0.049 (95% CI 0.040–0.057, see Fig. 2a). Heterozygous deletions are not reported in the study by Sievers et al. [37]. There was no significant heterogeneity regarding the prevalence of CDKN2A/B deletions among the included studies (I^2^ = 0%, *p* = 0.49). The distribution of CDKN2A/B deletions was further investigated by the stratified prevalence of homozygous or heterozygous CDKN2A/B deletions. The prevalence of homozygous CDKN2A/B deletion in meningiomas was 0.028 (95% CI 0.016–0.041, I^2^ = 73.6%, *p* = 0.013 see Fig. 2b). Data regarding the prevalence of heterozygous CDKN2A/B deletions were available in three studies. The pooled prevalence of heterozygous CDKN2A/B deletions was 0.024 (95% CI 0.018–0.031, see Fig. 2c). The prevalence of meningiomas with homozygous CDKN2A/B deletions significantly increased with the WHO grades (Fig. 2d–f): 0.002 (95% CI − 0.001–0.004) of WHO grade 1 meningiomas showed a homozygous CDKN2A/B deletion compared to 0.039 (95% CI 0.021–0.056) of WHO grade 2 meningiomas and 0.290 (95% CI 0.196–0.384) of WHO grade 3 meningiomas (*p* < 0.00001). No significant heterogeneity was found among the investigations reporting the distribution of CDKN2A/B status in WHO grade 1, 2, and 3 meningiomas.

### Reconstructed pooled progression-free survival curves and one-stage meta-analysis of the impact of CDKN2A/B on progression-free survival in meningioma

The reconstructed PFS curve and side-by-side comparisons with the original curves were conducted.

**Fig. 3:**
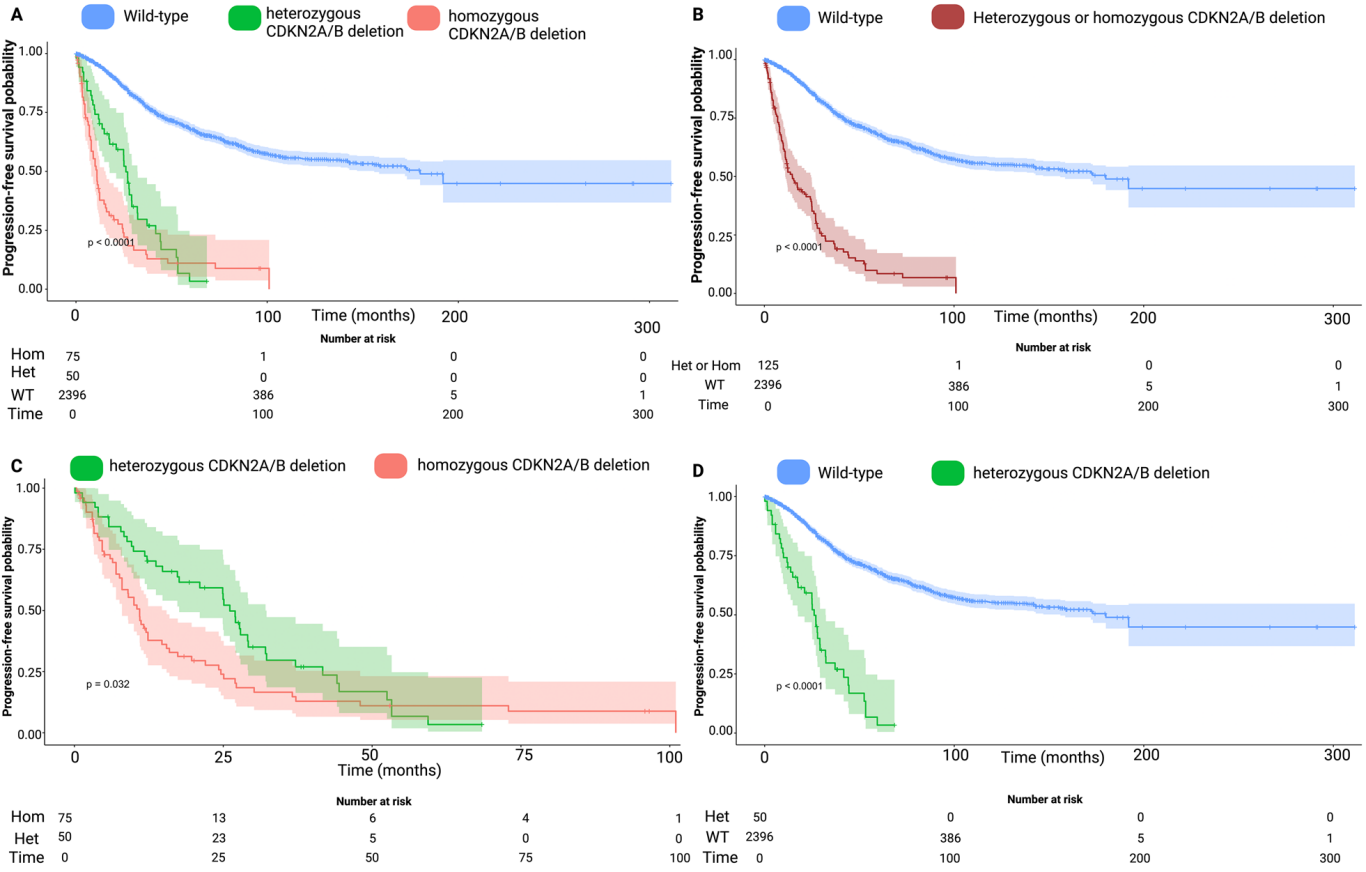
**a** Kaplan–Meier chart displaying probability of progression-free survival stratified by CDKN2A/B Wild-Type (blue), heterozygous (green) and homozygous CDKN2A/B deletions (red). The log-rank test (*p* < 0.0001) showed a significantly shortened time to meningioma progression in patients with either hetero- or homozygous CDKN2A/B deletions. The shadowed areas surrounding the curves display the confidence intervals **b** Kaplan–Meier chart displaying probability of progression-free survival stratified by CDKN2A/B Wild-Type (blue) and heterozygous or homozygous (brown) CDKN2A/B deletions. **c** Kaplan–Meier chart displaying probability of progression-free survival stratified by homozygous CDKN2A/B deletions (red) and heterozygous CDKN2A/B deletions (green). The log-rank test (*p* = 0.032) revealed a slightly significant difference regarding meningioma progression. **d** Kaplan–Meier chart displaying probability of progression-free survival stratified by CDKN2A/B Wild-Type (blue) and heterozygous (green) CDKN2A/B deletions. The log-rank test (*p* < 0.0001) revealed a significant shortened time to meningioma progression in those with a heterozygous CDKN2A/B deletion compared to those with a wild-type status. Het, Heterozygous; Hom, Homozygous; WT, Wild-type

All the reconstructed Kaplan–Meier plots and the published plots in each of the investigations were nearly identical, and the discrepancies in the number at risk tables were minor. The median (IQR) follow-up time of the reconstructed IPD was 36.7 months (17.0–73.0). The median time to meningioma progression in the CDKN2A/B wild-type arm was 180.0 (95% CI 145.7–214.3) months, whereas in those with a homo- or heterozygous CDKN2A/B deletion median PFS time was 14.8 (95% CI 10.0–19.6) months (*p* < 0.0001). The reconstructed Kaplan–Meier plots using IPD are further displayed by the stratification with CDKN2A/B wild-type, homozygous and heterozygous CDKN2A/B deletion (see Fig. 3a). The reconstructed PFS curve for the pooled population stratified by CDKN2A/B deletion (homo- or heterozygous) and CDKN2A/B wild-type is shown in Fig. 3b. Both meningioma patient groups with either hetero- or homozygous CDKN2A/B deletions had significantly shorter PFS times compared to those with CDKN2A/B wild-type. The median time to meningioma progression in the heterozygous CDKN2A/B deletion arm was 26.1 (95% CI 23.3–29.0) months, whereas in those with a homozygous CDKN2A/B deletion median PFS time was 11.0 (95% CI 8.6–13.3) months (*p* = 0.032, see Fig. 3c). However, even the heterozygous CDKN2A/B showed a significantly shortened time to meningioma progression (log-rank test: *p* < 0.0001) compared with the CDKN2A/B Wild-type arm (see Fig. 3d). The 12-, 24-, and 60-month PFS rates for CDKN2A/B wild-type meningiomas were 94.7%, 86.4%, and 67.8%. The group of meningioma patients with a homozygous CDKN2A/B deletion had 12-, 24-, and 60-month PFS rates of 41.1%, 25.8%, and 11.1%. The group of meningioma patients diagnosed with heterozygous CDKN2A/B deletions exhibited PFS rates of 70.2%, 55.9%, and 3.4% at 12, 24, and 60 months, respectively.

The estimated HRs and corresponding 95% CI of the included studies in the one-stage analysis are shown in Table 2. The Cox proportional hazards regression model yielded a significant HR of 6.8 (95% CI 5.5–8.5, *p* < 0.00001) for hetero- or homozygous CDKN2A/B deletions regarding meningioma progression. Homozygous CDKN2A/B deletion (HR: 8.4, 95% CI 6.4–11.0, *p* < 0.00001) was found to be significantly correlated with tumor progression. Furthermore, heterozygous CDKN2A/B deletion (HR: 5.5, 95% CI 4.0–7.6, *p* < 0.00001) was also significantly associated with meningioma progression.

**Table 2:** Estimated Hazard ratios (HR) and corresponding 95% confidence intervals regarding progression-free survival in the one-stage meta-analysis

References	Estimated HR	Estimated 95% CI
CDKN2A/B deletion (homozygous or heterozygous) versus wild-type		
Sievers et al. [37]	6.83	4.20–11.10
Driver et al. [6]	10.47	6.05–18.11
Wang et al. [46]	5.42	3.89–7.55
Khan et al. [16]	8.91	5.81–14.23
Entire IPD Cohort	6.83	5.50–8.47
Homozygous CDKN2A/B deletion versus wild-type		
Sievers et al. [37]	6.83	4.20–11.10
Driver et al. [6]	16.18	6.10–42-96
Wang et al. [46]	6.79	4.49–10.26
Khan et al. [16]	12.32	6.25–24.31
Entire IPD Cohort	8.35	6.36–10.95
Heterozygous CDKN2A/B deletion versus wild-type		
Driver et al. [6]	9.30	5.00–17.30
Wang et al. [46]	4.19	2.52–6.97
Khan et al. [16]	7.43	4.08–13.52
Entire IPD Cohort	5.52	3.99–7.62

### Two-stage meta-analysis

**Fig. 4:**
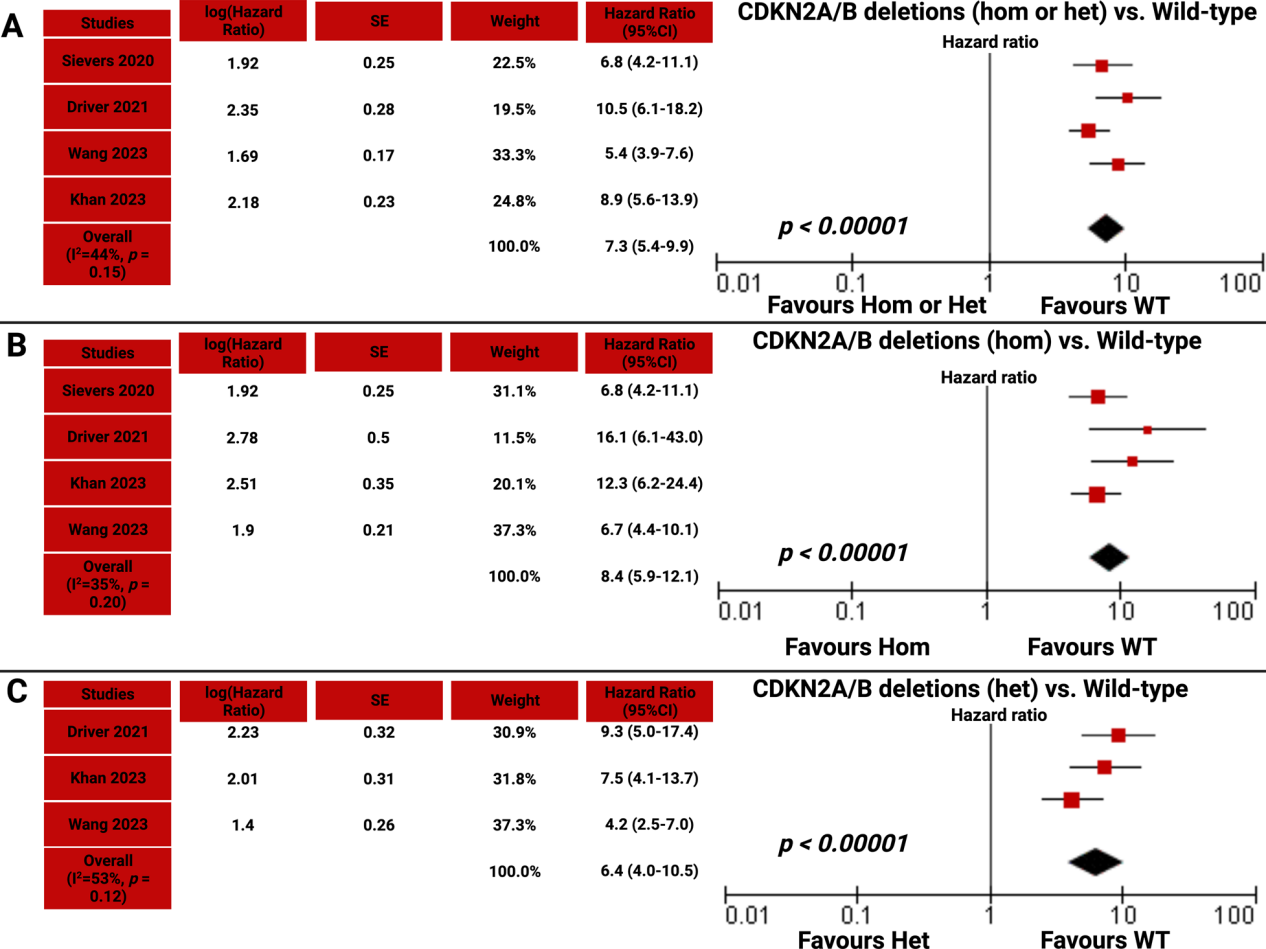
**a** Forest plot displaying log (hazard ratio), HR, and 95% CI estimates for PFS in a random effect model with the inverse variance method of the included studies [3, 12, 27, 36] evaluating any type (hetero- or homozygous) CDKN2A/B compared with wild-type. Risk of meningioma progression in patients with homozygous CDKN2A/B deletions compared with wild-type is shown in **b**. **c** Forest plot displaying the increased risk of meningioma progression in patients with heterozygous CDKN2A/B deletions compared with wild-type. X-axis locations of squares display the hazard ratio. The weight of the included studies is also presented. The diamond corresponds to the hazard ratio of the pooled data. The diamond corresponds to the hazard ratio of the pooled data. Het, Heterozygous; Hom, Homozygous; SE, Standard error; WT, Wild-type

In order to confirm the results of the one-stage analysis, a two-stage meta-analysis with a random effect was conducted to also validate the findings regarding the heterogeneity between the studies. Regarding PFS, the pooled HR of 7.3 (95% CI 5.4–9.9, *p* < 0.00001) confirms the significant findings of the single-stage meta-analysis that any type of CDKN2A/B deletions are strongly associated with shortened time to meningioma progression (see Fig. 4a). The assessment of PFS indicated minimal heterogeneity (I^2^ = 44%, *p* = 0.15). Further two-stage meta-analyses of patients with homozygous CDKN2A/B deletions compared with wild-type (see Fig. 4b) as well as patients with heterozygous CDKN2A/B deletions compared with wild-type (see Fig. 4c) were performed. Homozygous CDKN2A/B deletion was strongly associated with an increased risk of meningioma progression compared with wild-type (HR = 8.4, 95% CI = 5.9–12.1, *p* < 0.00001). Even heterozygous CDKN2A/B deletions were associated with an increased risk of meningioma progression compared with wild-type, too (HR = 6.4, 95% CI = 4.0–10.5, *p* < 0.00001). The assessment of PFS among patients with heterozygous CDKN2A/B deletions and CDKN2A/B wild-type indicated no significant heterogeneity (I^2^ = 53%, *p* = 0.12).

### Subgroup analysis of reconstructed PFS data with multiple common covariates

**Fig. 5:**
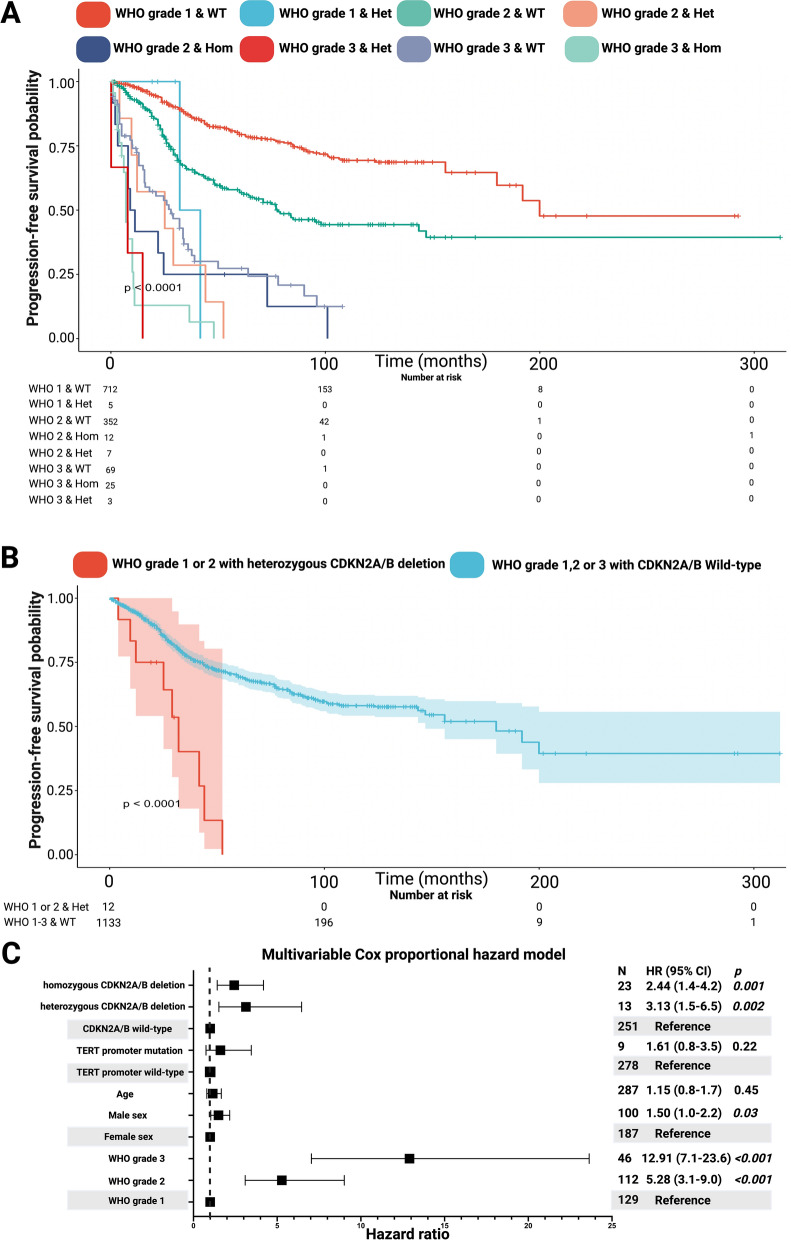
**a** Kaplan–Meier chart displaying probability of progression-free survival stratified by the combination of previous 2016 WHO grading combined with CDKN2A/B status (heterozygous, homozygous, and wild-type). **B** Kaplan–Meier chart displaying probability of progression-free survival stratified by WHO grade 1 or 2 meningiomas with a heterozygous CDKN2A/B deletion (red line) and WHO grade 1, 2 or 3 meningiomas with a CDKN2A/B wild-type status (turquoise line)** c** Forest plots from multivariable Cox regression analysis: Homozygous or heterozygous CDKN2A/B deletions, male sex, and WHO grade 2 or 3 are independent predictors of progression-free survival. The dashed line represents a hazard ratio of 1.0. *p* values in italics display statistically significant results. Het, Heterozygous; Hom, Homozygous; WT, Wild-type

The IPD of Khan et al. [16] and Sievers et al. [37] enabled a further analysis of PFS data stratified by both 2016 WHO grades and CDKN2A/B status. The previous findings of the one- and two-stage meta-analyses of the total IPD cohort indicated a potential prognostic importance of also patients with heterozygous CDKN2A/B deletions. The PFS data of the two mentioned studies were stratified by the combination of WHO grade and CDKN2A/B status. This subgroup analysis included the PFS data of 1185 patients. The median time to meningioma progression in those with CDKN2A/B wild-type status combined with WHO grade 1 was 199.9 months and in those combined with WHO grade 2 was 78.0 months. The previously as WHO grade 2 classified meningiomas despite a homozygous CDKN2A/B deletion had a median time to meningioma progression of 9.0 months (95% CI 3.6–14.4). However, patients with a WHO grade 2 meningioma with a heterozygous CDKN2A/B deletion (*n* = 7) had a median time to tumor progression of 25.1 months (95% CI 0.0–58.2 months), and those with a WHO grade 3 meningioma with a CDKN2A/B wild-type status had a median time to meningioma progression of 32.0 months (95% CI 25.1–38.9 months). The results of the further stratifications by WHO grade and CDKN2A/B status are shown in Fig. 5a. Furthermore, WHO grade 1 or 2 meningiomas with a heterozygous CDKN2A/B deletion were compared WHO grade 2 or 3 meningiomas with a CDKN2A/B wild-type status (see Fig. 5b). The median PFS time in the patients with WHO grade 1 or 2 meningiomas combined with a heterozygous CDKN2A/B deletion was 32.1 months (95% CI 22.7–41.5 months), and in those with a WHO 1, 2 or 3 meningioma combined with a CDKN2A/B wild-type was 180.0 months (95% CI 141.4–218.6 months), respectively (log-rank test: *p* < 0.0001).

To further investigate the prognostic role of heterozygous CDKN2A/B deletions regarding PFS, we analyzed 287 of those 1185 patients, who share the following common available covariates: Age, sex, WHO grade (1–3), CDKN2A/B status (homozygous deletion, heterozygous deletion, or wild-type), and TERT promoter status (mutation, wild-type). We performed multivariable Cox regression analysis of all factors potentially predicting PFS among these patients to determine independent risk factors of patients sharing common available covariates (see Fig. 5c). The multivariable analysis revealed the variables “WHO grade 2” (HR: 5.28, 95% CI 3.1–9.0, *p* < 0.001), “WHO grade 3” (HR: 12.91, 95% CI 7.1–23.6, *p* < 0.001), male sex (HR: 1.50, 95% CI 1.0–2.2, *p* = 0.03), “homozygous CDKN2A/B deletion” (HR: 2.44, 95% CI 1.4–4.2, *p* = 0.001), and “heterozygous CDKN2A/B deletion” (HR: 3.13, 95% CI 1.5–6.5, *p* = 0.002) to be independent predictors for a poor probability of progression-free survival.

### Bias and quality evaluation

**Fig. 6:**
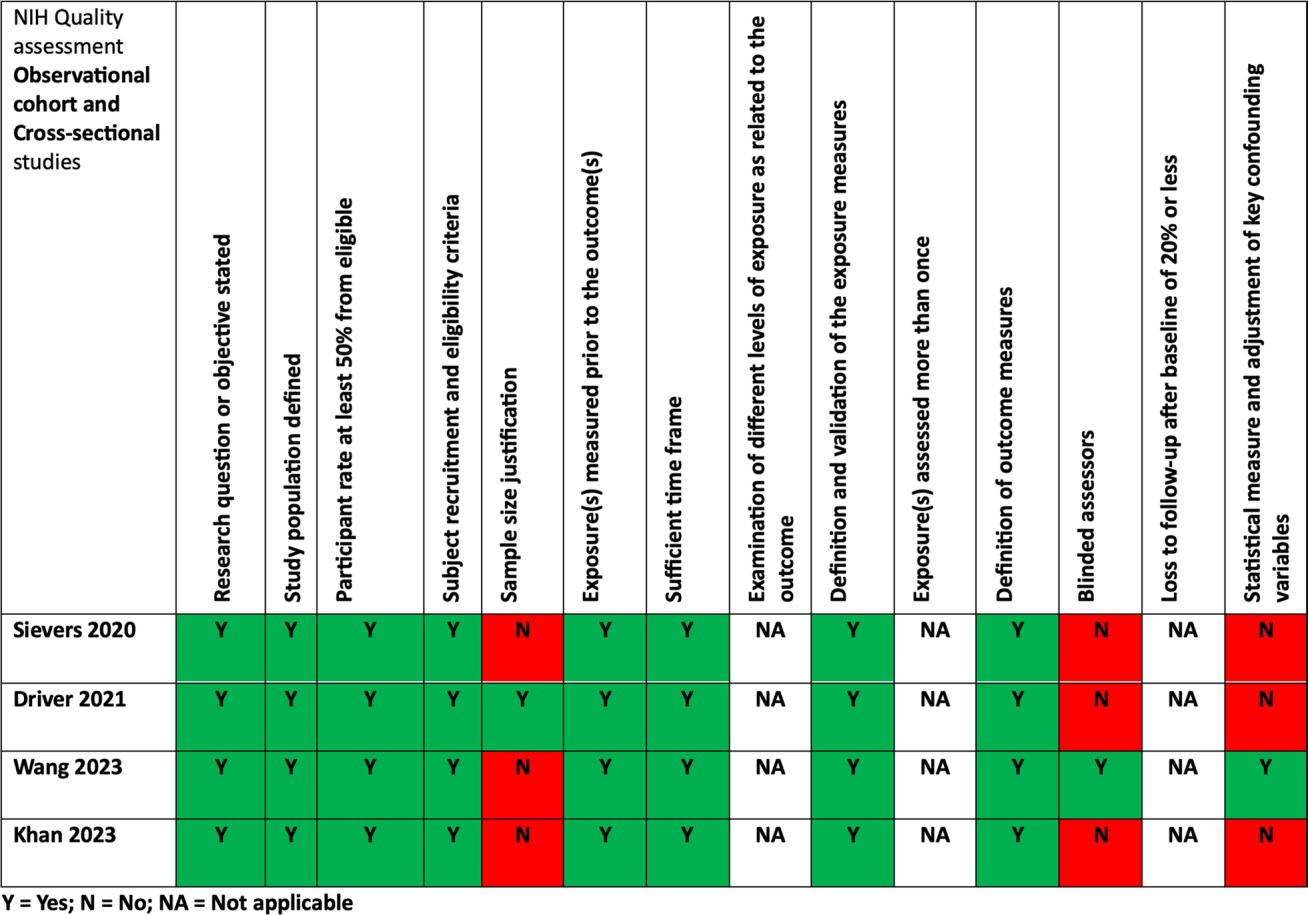
NIH-QAT assessment of included studies [6, 16, 37, 46]

The NIH-QAT tool was utilized to assess quality, resulting in favorable ratings for the included studies. The ratings for each of the 14 NIH-QAT domains can be found in Fig. 6. All studies were conducted as retrospective investigations and in the study by Wang et al. [46] the pathologist was blinded to the clinical data. Only the study by Wang et al. [46] provided results from a multivariable analysis. The major quality limitations pertained to the unclear sample size justification and limited control for confounding variables.

We assessed bias in the pooled prevalence of CDKN2A/B deletions and found no significant distortion across the included studies. The Egger’s regression test for funnel plot (see Additional file 1: Fig. S1) asymmetry showed that there was no evidence of publication bias (*p* = 0.083, z = 1.7315, intercept (b) = − 3.5278 (95% CI = − 4.1021–2.9534).

## Discussion

In recent years, advances in molecular genetic alterations have yielded new insights into the pathophysiology and clinical progression of meningiomas, particularly in relation to prognosis and outcome. The deletions of the CDKN2A/B gene and their impact on tumor progression, along with clinical outcomes, have also been demonstrated in other malignant CNS tumors, such as gliomas [21]. In the present meta-analysis, we investigated the prevalence of CDKN2A/B deletions with regard to the WHO grade and found that the proportion increases with the WHO grade. Generally, the overall prevalence of any type (heterozygous or homozygous) of CDKN2A/B deletions in meningiomas was 4.9%. Notably, nearly one third of the WHO grade 3 meningiomas have a homozygous CDKN2A/B deletion. The results indicate that CDKN2A/B plays an important role in malignant meningioma and cell proliferation. In our meta-analysis of IPD from 2521 meningioma patients, both heterozygous and homozygous CDKN2A/B deletions are a negative prognostic factor regarding the probability of PFS (Additional file 2: Fig. S2). Thus, homozygous as well as heterozygous CDKN2A/B deletions seem to be strong biomarkers for PFS as a potential surrogate marker for overall survival in WHO grade 2 or 3 meningiomas. Homozygous CDKN2A/B deletions are suggested to result in an uncontrolled cell cycle and the promotion of cell proliferation [35]. Hence, these CDKN2A/B (CDKN2A encodes for p14 & p16, CDKN2B encodes for p15) genes naturally code for three proteins suppressing the oncogenic CDK pathway [4]. The resultant functional loss of the proteins p14–p16 leads to a dysregulated cell cycle and other oncogenic pathways such as angiogenesis [10]. For instance, p14 inhibits the endothelial cell migration by the stimulation of the expression of tissue inhibitor of metalloproteinase 3 [49], whereas p16 inhibits angiogenesis by the regulator of vascular endothelial growth factors [10].

Based on the results of the present meta-analysis, we are able to demonstrate that both homozygous and heterozygous CDKN2A/B deletions significantly shorten PFS in a large-scale meningioma cohort. Thus, we can confirm the results of the individual included studies that strongly showed the role of homozygous CDKN2A/B deletions regarding PFS [6, 16, 37, 46]. Furthermore, the subgroup analysis of combining old WHO grading with CDKN2A/B status confirmed the present WHO grading regarding the importance of homozygous CDKN2A/B deletions because those with a previously diagnosed WHO grade 2 meningioma despite a homozygous CDKN2A/B deletion had a shorter time to meningioma progression compared with those with a WHO grade 3 meningioma combined with a CDKN2A/B wild-type status.

The distribution of CDKN2A/B deletion among the WHO grades suggests that this molecular alteration is associated with higher WHO grade and more aggressive meningiomas. Drawing from the present pooled dataset, the prevalence in WHO grade 1 meningiomas is 0.2%, while in WHO grade 2 meningiomas it is 3.9%. Conversely, nearly one-third (29.0%) of WHO grade 3 meningiomas exhibit a deletion in the CDKN2A/B gene. However, there are also large-scale investigations such as by Williams et al. [47], who investigated 377 sporadic meningiomas and found a prevalence of CDKN2A/B deletions in 8.0% of all cases.

Previous studies have investigated only either homozygous or heterozygous CDKN2A/B deletions regarding the impact on PFS. Therefore, we conducted distinct investigations of homozygous and heterozygous deletions independently, along with a pooled analysis of CDKN2A/B deletions compared with CDKN2A/B wild-type. This finding was also observed in the studies by Driver et al. [6] and Wang et al. [46], which compared the probabilities of PFS between 14 meningioma patients with heterozygous CDKN2A/B deletions and 24 patients with homozygous CDKN2A/B deletions. The pairwise log-rank test comparing the patients with heterozygous CDKN2A/B deletions compared with CDKN2A/B wild-type status showed that even a heterozygous CDKN2A/B deletion is of prognostic importance regarding PFS. This result was confirmed in the one- and two-stage meta-analysis. Furthermore, the small subcohort with a further stratification by the combination of WHO grading with CDKN2A/B status showed that WHO grade 1 or 2 meningiomas with a heterozygous CDKN2A/B deletion seem to have a poorer prognosis compared with WHO grade 1, 2 or 3 meningiomas with a CDKN2A/B wild-type status. Heterozygous CDKN2A/B deletion was also found to be independently associated with PFS in the multivariable Cox proportional hazard model. This finding is of paramount importance because heterozygous CDKN2A/B deletions are currently no diagnostic criteria for WHO grade 3 meningiomas according to the present WHO classification system [20]. Although we have identified a prevalence of only 2.4% with respect to heterozygous CDKN2A/B deletions, this particular finding warrants further investigation in larger cohorts. There may exist relatively small subcohorts of WHO grade 1 and 2 meningioma patients exhibiting heterozygous CDKN2A/B deletions, and these cases might be currently underestimated in terms of postoperative risk stratification for tumor progression. Additionally, there is a concern that current adjuvant therapy regimens might potentially undertreat those patients. Despite additional validation though both two-stage meta-analysis and multivariable Cox proportional hazard model, the findings have to be interpreted with caution due to the low proportional number of patients with heterozygous CDKN2A/B deletions.

The current literature is limited to only few studies reporting the impact of heterozygous CDKN2A/B deletions on PFS, which made it difficult to draw a precise conclusion. The methylation pattern plays a crucial role in the clinical applicability of CDKN2A/B as a biomarker. However, comprehensive exploration of the methylation pattern has been carried out in the study by Wang et al. [46]. They found increased CpG methylation at the CDKN2A gene, particularly in the gene body and at 3′ untranslated region (UTR) in those cases with an increased CDKN2A expression. However, this pattern was not seen in for CDK4. At the individual CpG level, they identified 14 different CpGs within the CDKN2A gene locus that displayed a significant correlation with CDKN2A mRNA expression. Nevertheless, previous investigations have found that apart from being deleted, CDKN2A hypermethylation as an epigenetic silencing is linked to unfavorable outcomes [18, 30]. Against this backdrop, it remains still unclear whether this hypermethylation serves as a regulatory mechanism contributing to increased expression or is merely a coincidental event within the broader context of elevated global methylation observed in aggressive meningiomas. Most of the studies included in this meta-analysis state explicitly or suggest that a genome-wide methylation array was used to obtain information about CDKN2A/B status as described in Capper et al. [3]. Although not explicitly stated it is likely safe to assume that tissue samples were fixed and processed using standard methods, thus resulting in comparable quality standards [2, 3]. However, it also has to be reminded that the prevalence of CDKN2A/B deletions could be also influenced by a sampling bias. The included studies did not allow a further stratification of the data regarding the extent of resection in those patients with available CDKN2A/B status and PFS data [5]. For instance, it might be possible that in a subtotally resected meningioma the analyzed tumor tissue does not necessarily contain the hotspot region with the maximum aggressive behavior showing CDKN2A/B deletions. Further large-scale trials of WHO grade 2 and 3 meningiomas investigating heterozygous CDKN2A/B deletions with consideration of extent of resection as well as adjuvant radiation therapy regimes have to be performed.

Only one of the included studies in the present meta-analysis reports data on the anatomical localization of meningiomas. Hence, we could not stratify the prognostic impact of CDKN2A/B deletions regarding PFS by the anatomical locations. Anatomical location of meningioma is also an important factor influencing the biological behavior as well as the probability of a gross total resection. For instance, it is known that the extent of resection has a lower impact regarding PFS in skull base meningiomas compared with non-skull base meningiomas [42]. Furthermore, several typical meningioma locations have distinct related genomic markers (e.g., anterior skull base: SMO, AKT1E17K, TRAF7; central skull base: AKT1, KLF4, TRAF7, POLR2A, Convexity: NF-2, LOH chromosome 22, BAP1) [29]. Furthermore, the MIB-1 labeling index as well as the density of macrophage infiltrates significantly differs between skull base and non-skull base meningiomas [44]. Since transcriptomics is distributed differently in brain areas, it is also likely that CDKN2A/B expression is different in different brain areas [47]. Moreover, meningiomas are known to harbor significant intratumoral heterogeneity which range from the morphological to the genetic and epigenetic levels [1, 24, 31]. Several studies suggest that spatial heterogeneity is likely due to subclonal progression of acquired chromosomal abnormalities and driver events such as TERT promoter mutations [1, 14, 22]. To our knowledge, no direct experimental evidence of heterogeneous loss of CDKN2A/B is available. However, several lines of evidence support the notion that CDKN2A/B loss might represent a subclonal event occurring late in tumorigenesis and thus present with a heterogeneous spatial distribution [22, 46].

A recent multicenter cohort study of 103 WHO grade 3 meningiomas across North America and Europe demonstrated that only Age ≥ 65 years and male sex are independent predictors of progression-free survival in WHO grade 3 meningiomas [40]. Hence, the classic prognostic factors such as extent of resection and adjuvant radiotherapy seem to be not entirely transferable to WHO grade 3 meningiomas [8]. Against this backdrop, it is of paramount importance to remind that the prevalence of CDKN2A/B deletions was 29.0% among the WHO grade 3 meningiomas in the present meta-analysis. Hence, this molecular genetic alteration should be strongly considered in the clinical care and future tailored therapy regimes for WHO grade 3 meningioma patients might benefit from these data. Future studies will have to focus on the prognostic impact of heterozygous CDKN2A/B deletions because the present data suggests that both types of CDKN2A/B deletions should have diagnostic and therapeutic consequences. The exact molecular reasoning for heterozygous deletion to be as important as homozygous deletions remains unclear from our meta-analysis. Irrespective of CDKN2A/B loss, the transcriptional level of CDKN2A/B harbor prognostic significance in meningioma [46]. Recent studies demonstrate that contrary to the expected effect of CDKN2A/B loss, elevated levels of CDKN2A mRNA are associated with lower PFS and more malignant molecular and methylation classes in CDKN2A/B intact/wild-type meningiomas [46]. These findings suggest different molecular mechanisms of tumorigenesis with potential therapeutical relevance. However, meningiomas of the malignant class (Molecular Group 4) show the most biologically aggressive behavior and CDKN2A/B might not provide additional prognostic information in this group [46]. From a pharmacological point of view, selective inhibitors of CDK4 and CDK6 such as Palbociclib might be a promising strategy for these tumors. A preclinical in vitro and in vivo model with patient-derived meningioma cells treated with palbociclib and radiation revealed that palbociclib might be used to treat meningioma patients with reduced or absent p16 expression [13]. CDK4/6 inhibitors are already approved for use in hormone receptor-positive breast cancer combined with endocrine treatment [25]. Currently, there is an ongoing multi-arm study (NCT02523014) investigating abemaciclib in recurrent meningioma patients with alterations in the CDK pathway or altered NF2 gene. However, CDK4/6 inhibitors might be not efficacious for those with a retinoblastoma-deficient tumor [13]. Furthermore, it has been demonstrated that meningiomas with homozygous CDKN2A/B deletions frequently harbor the loss of the methylthioadenosine phosphorylase (MTAP), which is in close proximity of the gene loci [33]. MTAP immunohistochemistry has been demonstrated to be a surrogate marker for homozygous CDKN2A/B deletions and might highlight patients potentially benefiting from novel therapies inhibiting the promotion of DNA damages and mitotic dysfunction in meningioma cells [15]. Tumors with MTAP loss are vulnerable for therapies inhibiting the methionine adenosyltransferase 2α because of the reduction of S-adenosylmethionine levels [15]. Currently there is a phase I clinical trial investigating the MAT2A inhibitor AG-270 in advanced solid tumors or lymphomas with homozygous deletions of CDKN2A/B or MTAP (NCT03435250). However, a brain penetrating MAT2A inhibitor was recently developed and awaits further preclinical tests for CNS pathologies with CDKN2A/B deletions [17].

The present meta-analysis has several limitations. First, the studies included in this meta-analysis reported patient data prior to the most recent WHO classification of central nervous system tumors [20]. Second, the individual patient data cannot be further stratified by important stratified by TERT promoter status, extent of resection, and adjuvant radiation therapy. However, we were able to perform a subgroup analysis using the IPD of two studies to demonstrate the importance of both hetero- and homozygous CDKN2A/B deletions in the setting of the 2016 WHO grading [16, 37]. Third, sampling bias due to a potential high variability of the extent of resection might also influence the prevalence of detected CDKN2A/B deletions and the PFS analysis [5]. To date, various methods assess CDKN2A/B deletions, including single-nucleotide polymorphism (SNP) micorarrays, next-generation sequencing (NGS), DNA-based methylation studies, and fluorescent in situ hybridization (FISH). Accuracy highly depends on assay types and differs in genomic resolution. Hence, some studies combine CDKN2A and CDKN2b assessment. NGS include targeted panels and whole genome sequencing, whereas methylation arrays use HumanMethylation450 (450 k) and MethylationEPIC (850) arrays. Hence, there can be inter-laboratory differences regarding the methods and the findings. Nevertheless, three of the four included studies used either the 850 k or 450 k arrays of Illumina (Illumina, San Diego, CA, USA) to assess the methylation profiling and copy numbers [16, 37, 46], and one study used the Agilent SurePrint G3 1 × 1 microarray (Agilent, Santa Clara, CA, USA) [6]. However, FISH detects deletions with 20–30% tumor cell thresholds, but might miss smaller deletions. Immunohistochemical diagnostic workup by p16 staining as a surrogate marker varies in sensitivity and specificity, which might offer poorer prognostic insight than molecularly determined CDKN2A/B loss. Most of the studies included in the present meta-analysis either state explicitly or suggest that CDKN2A/B loss was inferred from copy number variations as described in Capper et al. [3]. Our analysis thus indirectly confirms that homozygous and heterozygous CDKN2A/B deletions as determined in the included studies is sufficiently robust, to allow for the identification of an independent predictor of PFS in meningioma. Nevertheless, the availability of genome-wide methylation analysis provides additional information that harbors prognostic significance. For instance, it has been shown that hypermethylation in the gene body or 3’UTR region of the CDKN2A gene are associated with higher CDKN2A expression and hence with a worse prognosis in meningioma as well as other malignancies such as colorectal cancer [36, 46]. Hence, future investigations will have to focus on CDKN2A methylation transcriptional data. All in all, there is there is also the strong need to compare those methods evaluating CDKN2A/B deletions themself regarding their prognostic value in terms of PFS and OS probability [48].

In conclusion, the present meta-analysis is the first investigation using reconstructed individual patient data to analyze the impact of heterozygous or homozygous CDKN2A/B deletions on progression-free survival in meningiomas. The results indicate that both homozygous and heterozygous CDKN2A/B deletion significantly worsen PFS time. Heterozygous CDKN2A/B deletion seem to have a nearly identical poor prognostic impact and should be further investigated to provide those patients an adequate therapy. Furthermore, nearly one third of all WHO grade 3 meningiomas have CDKN2A/B deletions, which necessitates further research on markers identifying patients who might benefit from CDK4/6 inhibitors. These findings might provide information for future prospective clinical studies investigating targeted drug therapies for high-grade meningiomas with CDKN2A/B gene alterations.

### Supplementary Information

**Additional file 1:** Funnel plot assessment for publication bias from pooled prevalence of CDKN2A/B deletions. A symmetric funnel plot emerges when highly precise studies cluster near the pooled meta-analysis estimate at the apex of the funnel, whereas less precise studies exhibit effect sizes evenly distributed both below and above the pooled estimate. The presence of asymmetry in a funnel plot allows for the measurement of publication or reporting bias. Consequently, statistical testing of a funnel plot provides insight into whether the reported effect in the literature is biased or systematically skewed in a specific direction.

**Additional file 2:** Illustrative visual summary of the main findings of the present investigation.

## Data Availability

The original contributions presented in the study are included in the manuscript. Further inquiries can be to the corresponding authors of the article.
