# Supplementary figures and images for: CDKN2A/B deletions are strongly associated with meningioma progression: a meta-analysis of individual patient data
Source: Acta Neuropathol Commun. 2023 Nov 28;11:189. doi: 10.1186/s40478-023-01690-y (PMC10685484; doi:10.1186/s40478-023-01690-y)

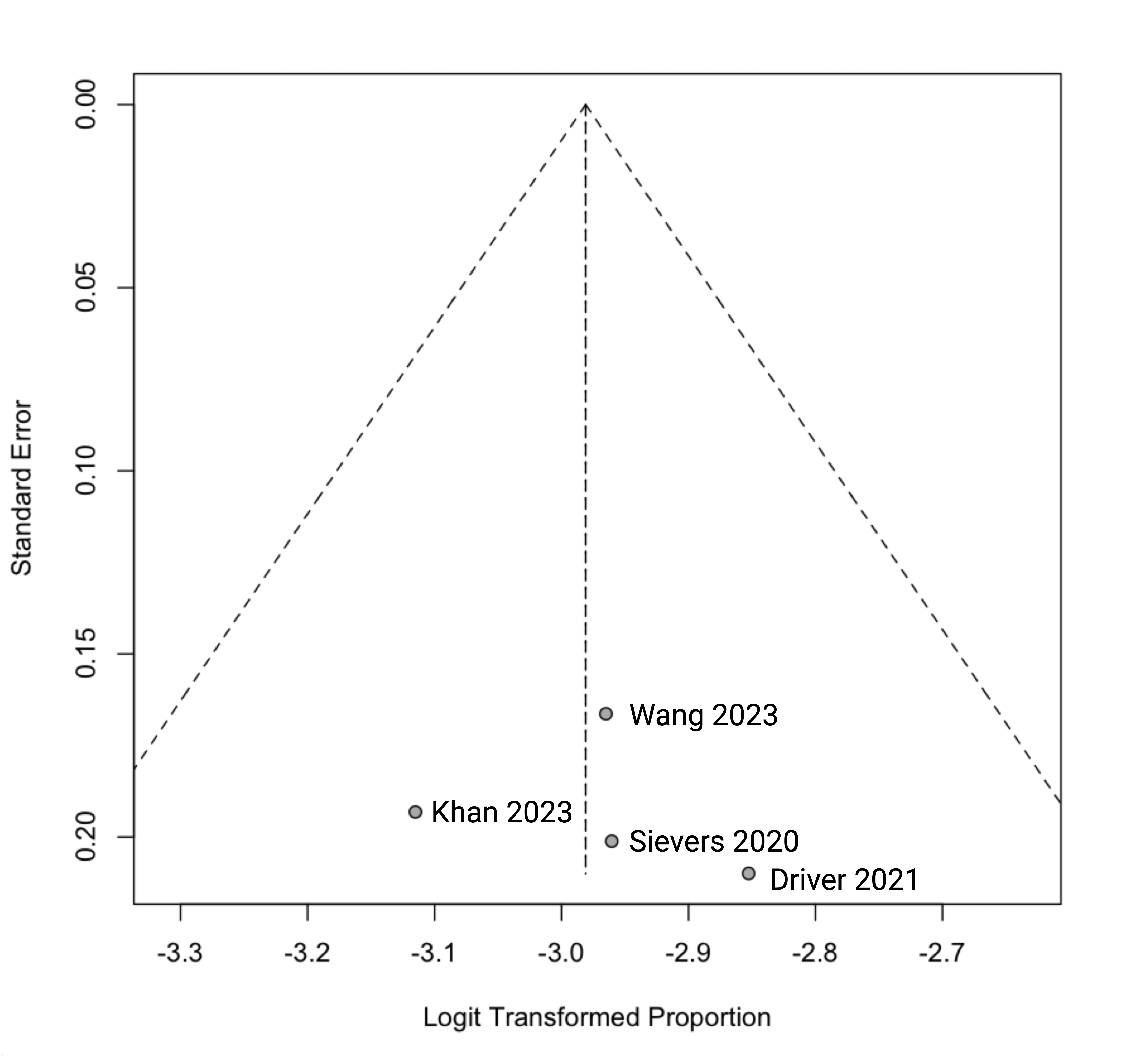

Supplement: Supplementary file 1 — Additional file 1: Funnel plot assessment for publication bias from pooled prevalence of CDKN2A/B deletions. A symmetric funnel plot emerges when highly precise studies cluster near the pooled meta-analysis estimate at the apex of the funnel, whereas less precise studies exhibit effect sizes evenly distributed both below and above the pooled estimate. The presence of asymmetry in a funnel plot allows for the measurement of publication or reporting bias. Consequently, statistical testing of a funnel plot provides insight into whether the reported effect in the literature is biased or systematically skewed in a specific direction. [file 40478_2023_1690_MOESM1_ESM.png]

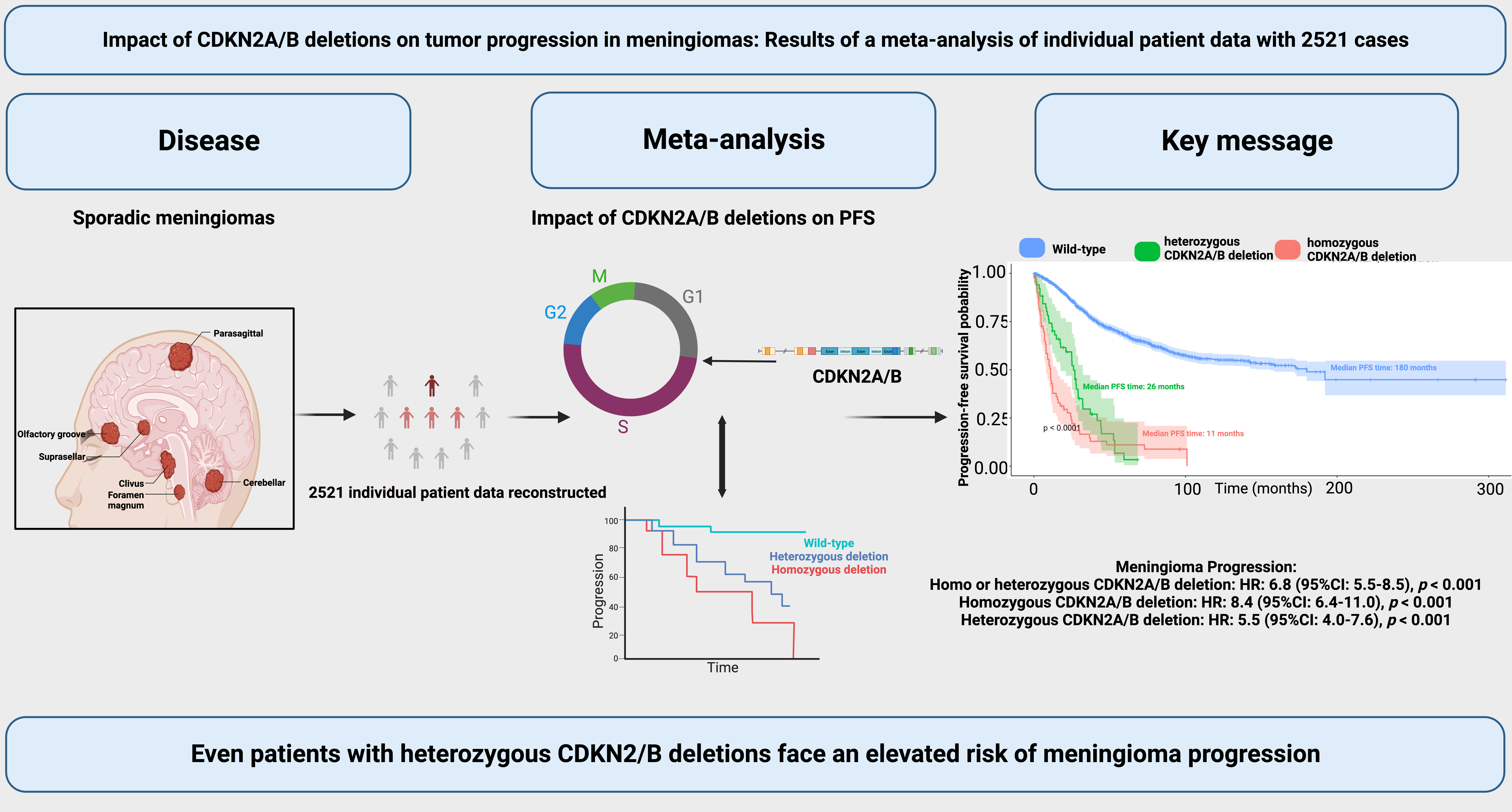

Supplement: Supplementary file 2 — Additional file 2: Illustrative visual summary of the main findings of the present investigation. [file 40478_2023_1690_MOESM2_ESM.png]
